# An Alternative In Vitro Propagation Protocol of *Cannabis sativa* L. (Cannabaceae) Presenting Efficient Rooting, for Commercial Production

**DOI:** 10.3390/plants11101333

**Published:** 2022-05-18

**Authors:** Kostas Ioannidis, Ioanna Tomprou, Vangelis Mitsis

**Affiliations:** 1 Laboratory of Sylviculture, Forest Genetics and Biotechnology, Institute of Mediterranean and Forest Ecosystems, Hellenic Agricultural Organization “Demeter”, 11528 Athens, Greece; 2 Ekati Alchemy Lab SL, Carretera Barcelona 11, 08180 Moia, Spain; jtomprou2@gmail.com (I.T.); ekatimed@gmail.com (V.M.)

**Keywords:** hemp, in vitro culture, shortening in vitro process, vented and non-vented vessels, rooting peat-based sponges

## Abstract

An alternative in vitro propagation protocol for medical *Cannabis sativa* L. cultivars for pharmaceutical industrial use was established. The aim of the protocol was to reduce the culture time, offering healthy and aseptic propagating material, while making the whole process more economic for industrial use. The propagation procedure was performed using plastic autoclavable vented and non-vented vessels, containing porous rooting fine-milled sphagnum peat moss-based sponges, impregnated in ½ Murashige and Skoog liquid growth medium, supplemented with indole-3-butyric acid (IBA) at various concentrations (0, 2.46, 4.92, and 9.84 µM) or by dipping nodal cuttings into 15 mM IBA aqueous solution. The highest average root numbers per cutting, 9.47 and 7.79 for high cannabidiol (H_CBD) and high cannabigerol (H_CBG) varieties, respectively, were achieved by dipping the cuttings into IBA aqueous solution for 4 min and then placing them in non-vented vessels. The maximum average root length in H_CBD (1.54 cm) and H_CBG (0.88 cm) was ascertained using 2.46 μM filter sterilized IBA in non-vented vessels. Filter-sterilized IBA at concentrations of 2.46 μM in vented and 4.92 μM in non-vented vessels displayed the maximum average rooting percentages in H_CBD (100%) and H_CBG (95.83%), respectively. In both varieties, maximum growth was obtained in non-vented vessels, when the medium was supplemented with 4.92 μM filter-sterilized IBA. Significant interactions between variety and vessel type and variety and IBA treatments were observed in relation to rooting traits. Approximately 95% of plantlets were successfully established and acclimatized in field. This culture system can be used not only for propagating plant material at an industrial scale but also to enhance the preservation and conservation of *Cannabis* genetic material.

## 1. Introduction

*Cannabis sativa* L. is a widespread species that is cultivated worldwide in wide-ranging habitats areas outside its natural range. It is considered one of the oldest domestic and cultivated plants in the history of mankind [1]. Historical evidence bears testament to its medicinal properties, as recorded by many ancient civilizations [2]. Its first appearance is believed to have occurred in central Asia around 5000 B.C. [3] or even earlier [4]. In particular, according to Schultes et al. [1] and Merlin [5], hemp spread from the temperate and alpine foothills of the Himalayas 10,000 years ago.

*C. sativa* L. is a crop species that has multiple roles. It has been cultivated for industrial, nutritional, and, recently, for mainly medicinal purposes [6,7,8]. For centuries, hemp stems have been used for fibers (mats, shoes, cloth, and ropes) and its seeds have been used for oil production [7,9]. Moreover, hemp seeds are an excellent source of omega-3 and omega-6 fatty acids, as well as other nutritious oil and proteins [6,10,11]. Recently, the stem tissues have begun to be used in the manufacture of bioplastics and concrete-like material [6,12] and moreover for high-performance composite applications [13].

In recent decades, there has been a resurgence of interest in the use of bioactive compounds from natural sources, such as hemp, with constantly increasing demand [14]. Hemp flowers primarily and its leaves incidentally produce about 545 bioactive secondary metabolites [15]. The use of these substances, such as terpenoids, flavonoids and phytosterols [16], and alkaloids and glycoproteins [17], as well as a special class of terpenophenolic compounds, the cannabinoids [18], is constantly increasing [15] and the majority of them have medicinal properties. Among the numerous cannabinoids existing in hemp flowers, the most studied phytocannabinoids in relation to their therapeutic uses are the intoxicating Δ^9^-tetrahydrocannabinol (Δ^9^-THC), a promising medicinal compound for the treatment of various diseases [19] with well-known medicinal effects [20]; cannabidiol (CBD), which has several proven pharmacological properties [21,22]; and cannabigerol (CBG) [23,24,25,26] for its potential remedial effects.

Cannabinoid production through the exploitation of natural resources is constantly increasing [9,14], due to these plants’ apparent health, nutritional, and mostly medicinal properties [6,7,8,10,11]. For instance, *C. sativa* L. sales in the United States are expected to rise from USD 8 billion, as recorded in 2018, to over USD 40 billion by 2025 [27]. This imposes a need to detect and preserve genetic resources of *C. sativa* L. varieties that are rich in bioactive secondary metabolites [9] and, importantly, to supply the international market with adequate quantities from reliable sources. In vitro mass propagation, apart from being economical and fast, can also produce efficient quantities, with desirable chemical profiles, which is thus attractive for pharmaceutical industrial applications. Moreover, the optimization of in vitro propagation protocols of *C. sativa* L. varieties and their integration into the commercial production process is another crucial prerequisite [28].

In vitro propagation techniques offer efficient multiplication yields of disease-free *C. sativa* L. plants at a commercial scale. Generally, such techniques eliminate cultivation space and reduce production costs and time [29,30]. The products of these processes display genetic and phenotypic uniformity [9,31,32,33] in terms of their morphological traits, which include some of the major commercially important traits [34]. Moreover, the method has tremendous potential for genetic transformation by modifying both the genetic information and the regulation of those genes responsible for the production of valuable biologically active substances [30].

Several innovative systems and protocols were used in order to make the use of in vitro plant tissue culture an even more economical and time-saving vegetative propagation methodology. Traditionally, the conventional in vitro micropropagation procedure contains four stages, i.e., culture establishment, proliferation, rooting of shoots, and acclimatization [35]. According to Debergh and Maene [36], in vitro rooting costs represent 35–75% of the total process. Gradually, labor costs concerning conventional micropropagation, with the exception of culture establishment, have reached approximately 60% [37]. Cuttings, in the first three phases, are enclosed in vessels with passive gas exchange, under aseptic conditions. An upturn in tissue culture led to the onset of the photoautotrophic micropropagation technique, in which chlorophyllous explants were grown under CO_2_-rich conditions [29,38]. Moreover, culturing explants in vessels with gas-permeable film as enclosures, combined with the use of rockwool multi-blocks as a substrate were found to be suitable for the development of some plant species [39,40,41]. Photoautotrophic micropropagation on rockwool blocks as a substrate was efficient for *C. sativa* L. cultivation as well [42]. An alternative that has further improved on the performance of the vitro process is the use of a double-phase culture system (semi-solid medium with a layer of liquid medium on the top), in which shooting and rooting are performed simultaneously [43,44]. Other approaches include the use of bioreactors, which improve the physiological state of the explants. Such systems are commercially available, such as automated temporary immersion (RITA ^®^) [45] and Plantform ^TM^ [46] bioreactors, the rocker system [47,48], as well as others [49,50]. Hence, an improvement in in vitro plant tissue culture techniques for mass propagation is highly desirable [28]. With the aim of simplifying the propagation method, eliminating the use of expensive bioreactors, using the lowest possible amount of nutrient media and hormones for rooting, and diminishing root disturbances, we attempted to combine the advantages of the older methods used by Tanaka et al. [39] and Nagae et al. [40], while also integrating recent applications such as those of Teixeira da Silva et al. [38], Kodym and Leeb [42], and Vidal and Sanchez [49].

**Figure 1:**
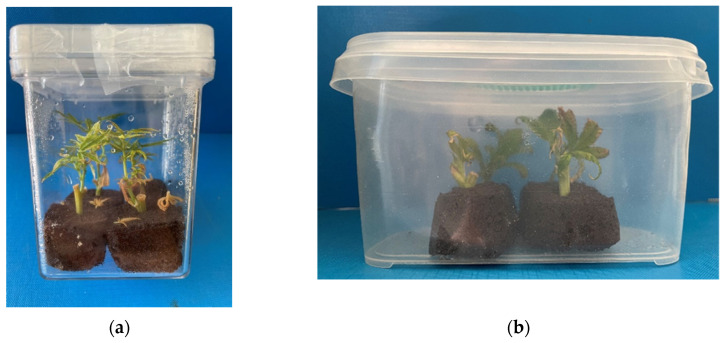
New growth of established cuttings in rooting peat-based sponges in non-vented (**a**) and vented (**b**) vessels.

With the objective of increasing the efficiency of the propagation process in terms of time and cost-savings, as well as the genetic conservation of the selected varieties that are rich in CBD, CBG, and THC, we have successfully developed an efficient alternative in vitro propagation protocol for mass production without compromising the chemical quality of the plants. In the present study, we investigated in vitro cutting disinfestation, culture establishment, and root induction, as well as the acclimatization of the in vitro-propagated plantlets using pet-based sponges as a substrate, impregnated in liquid medium (Figure 1).

## 2. Results

### 2.1. In Vitro Cutting Rooting

**Figure 2:**
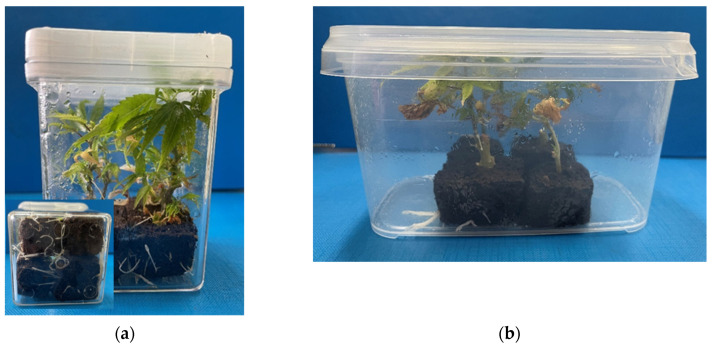
Root formation of cuttings in non-vented (**a**) and vented (**b**) vessels.

The disinfestation protocol of *C. sativa* L. cuttings was effective at a percentage of 87%. Root formation (Figure 2), i.e., the number of roots and root length, and cutting growth were significantly affected by the concentration of plant growth regulators, the vessel type, and the variety (Table 1 and Table 2). The number of roots per cutting was influenced by the variety (*p* ≤ 0.05), the vessel type (*p* ≤ 0.001), and the IBA treatments (*p* ≤ 0.001). Throughout the experiment, significant interactions between variety and vessel type (*p* ≤ 0.01) and variety and IBA treatments (*p* ≤ 0.001) were observed. The average root length per cutting was influenced by the variety (*p* ≤ 0.001), the vessel type (*p* ≤ 0.001), and the IBA treatments (*p* ≤ 0.001). However, significant interactions were only detected between the variety and the IBA treatments (*p* ≤ 0.001). The rooting percentage was affected only by the IBA treatments (*p* ≤ 0.001) and no interactions between the factors were identified. The effects of plant growth regulator concentrations on the average number and length of roots as well as the root formation frequency of the *C. sativa* L. varieties’ cuttings are presented in Table 1 and Table 2, respectively.

Root initiation of in vitro-propagated cuttings was commenced during the third week of culture, depending on the rooting treatment. Statistically significant differences (*p* ≤ 0.05) in the average number and length of roots per cutting and root formation frequency among different treatments were observed within each variety. Both H_CBD and H_CBG showed the same pattern in the studied traits in terms of the studied rooting traits.

The dipping treatment clearly outweighed the other treatments in terms of its effect on the average root number per cutting. Concerning H_CBD for non-vented vessels, the highest average root numbers per cutting were observed when dipping the cutting tips in 15 mM IBA aqueous solution for 4 and 2 min, respectively. These showed statistically significant differences (*p* ≤ 0.05) with the values of all the other treatments. Similarly, for vented vessels, the highest average root number per cutting was observed in the same treatment as above. Again, these were statistically significantly different (*p* ≤ 0.05) from the majority of the other treatment’s values. For vented and non-vented vessels, dipping treatments presented similar results. The treatments in which the culture medium was enhanced by IBA were less effective, showing comparable values (Table 1). Compatible results were found regarding H_CBG. For vented and non-vented vessels, the highest average root numbers per cutting were observed in dipping treatments, which were statistically significant different (*p* ≤ 0.05) compared to the majority of the other treatments. When dipping cuttings’ base tips in 15 mM IBA aqueous solution for 4 min, the maximum average number of roots was achieved in non-vented and vented vessels, respectively (Table 2).

Regarding the average root length, the addition of IBA via syringe filter-sterilization in the medium showed the highest values, presenting significant differences (*p* ≤ 0.05) compared to other treatments, regardless of the vessel type. For the H_CBD, the best average length of roots was obtained in non-vented vessels when 2.46 μΜ of IBA was added in the medium. The addition of 4.92μM resulted in the second-highest value. Τhe same trend was present in vented vessels, in which the best results were obtained via the addition of 2.46 μM. The last three treatments presented statistically significant differences (*p* ≤ 0.05) from the majority of the other treatments (Table 1). Concerning H_CBG, the results were repeatable to some extent. Filter-sterilization of IBA using a syringe led to best results. Specifically, in non-vented vessels, the highest average root length per cutting was obtained with the addition of 4.92 μM IBA, compared to the relevant cutting in vented vessels when 2.46 μM IBA was added. The dipping treatment using 15 mM IBA for 2 min presented non-statistical differences from both previous treatments (Table 2). In general, cuttings in non-vented vessels performed better compared to those in vented vessels (Table 1 and Table 2).

In regard to the average rooting percentage, the results were more uniform, as several treatments showed comparable results. Related to H_CBD, when supplementing the medium with 2.46 μM IBA via filter sterilization in vented vessels, all cuttings formed roots. In the same set of treatments, the second highest root formation frequency was observed using filter-sterilized IBA at a concentration of 4.92 μM and dipping the cuttings for 2 min (Table 1). The use of non-vented vessels led to relatively lower percentages. The best rooting formation frequency was obtained using 4.92 μM filter-sterilized IBA. Adding IBA via filter sterilization compared to dipping treatments did not show significant differences (*p* ≤ 0.05). Regarding H_CBG, the highest average rooting percentage was achieved in non-vented vessels, when the medium was supplemented generally with 4.92 μM IBA. For non-vented vessels, generally, adding IBA into the MS medium showed the highest average rooting percentage compared to the dipping treatments (Table 2). The results for vented vessels were somewhat different. The highest rooting formation frequency was obtained using 4.92 μM IBA, which was sterilized along with the medium or via the dipping treatment with 15 mM IBA for 2 min. On average, supplementing media with filter-sterilized IBA compared to dipping treatments resulted in non-significant differences (*p* ≤ 0.05).

In all treatments, controls showed the lowest values, except in the one concerning the average number of roots per cutting, in the case of the H_ CBD cuttings established in the autoclaved mixture of ½ MS medium and supplemented with 2.46 μM IBA in vented vessels.

### 2.2. In Vitro Cutting Growth

Shoot elongation was achieved after at least three weeks of culture, depending on the applied treatment and variety (Figure 2). Within each variety, statistically significant differences (*p* ≤ 0.05) in the average lengths of shoots among different treatments were observed. The average length of shoots was influenced by the variety (*p* ≤ 0.01) and the vessel type (*p* ≤ 0.001) and no significant interaction was observed either between the variety and the vessel type nor the variety and the IBA treatments. The effects of the concentrations of plant growth regulators on the growth of the *C. sativa* L. varieties’ cuttings are presented in Table 3.

Regarding H_CBD and the non-vented vessels, the maximum growth was achieved when supplementing the medium with 4.92 μM IBA via filter sterilization. Dipping of the cuttings into 15 mM IBA aqueous solution for two minutes led to the second-highest growth. The use of an autoclaved mixture of medium and 4.92 μM IBA did not lead to statistically different result compared with the latter treatment. The same pattern was observed in vented vessels. Adding 4.92 μM IBA via filter sterilization in the medium or autoclaving the mixture of the medium and 4.92 μM IBA led to the best growth values. In vented vessels, filter sterilization of the IBA presented the best results. In contrast, the dipping treatment showed the lowest growth values, comparable to controls, which also did not show statistically significant differences (*p* ≤ 0.05).

The results of obtained for H_CBG were comparable to those of H_CBD. In non-vented vessels, the maximum growth was achieved by supplementing the medium with 4.92 μM IBA via filter sterilization. The second- and third-highest growth values were obtained when the autoclaved mixture of medium + 4.92 μM IBA and dipping treatment of the cuttings into 15 mM IBA for two minutes were used, respectively. These treatments did not show statistically significant differences (*p* ≤ 0.05). Concerning vented vessels, we observed corresponding results with the previous ones. Supplementing the medium with 4.92 μM filter-sterilized IBA led to the best growth values. The best subsequent results were achieved when cuttings were dipped into 15 mM IBA for two minutes or placed into an autoclaved mixture of medium supplemented with 4.92 μM IBA. None of the latter three treatments showed statistically significant differences (*p* ≤ 0.05). Generally, in vented vessels, filter sterilization of the IBA presented the best results. Conversely, dipping treatments showed the lowest growth values, which were statistically equal (*p* ≤ 0.05), although in comparison with their controls, they showed statistically significant differences.

In all treatments, controls again showed the lowest growth values, again with the exception of the H_CBD cuttings which were dipped in 15 mM IBA for four minutes in vented vessels.

### 2.3. Acclimatization

**Figure 3:**
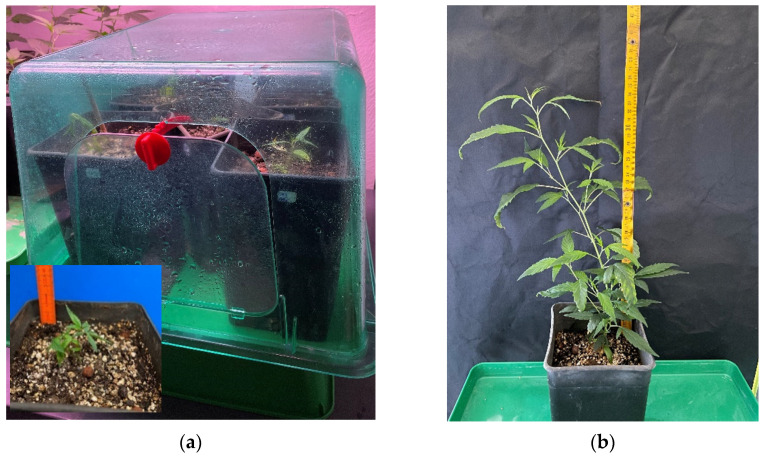
Well-rooted cuttings placed in peat–perlite mixture for acclimatization (**a**) and fully acclimatized *Cannabis sativa* L. plant (**b**).

Vigorous cuttings with well-developed roots, i.e., visible roots protruding from the sponges, were successfully transplanted in plastic pots containing a sterile peat:perlite mixture that was placed in mini greenhouses (Figure 3). The cuttings acclimatized relatively easily to ex vitro conditions (95%) with low losses. New shoots and growth were observed within 2 weeks. The rooted cuttings were acclimatized within three to four weeks. The acclimatized plantlets were transplanted to flowerpots and placed indoors under controlled environmental conditions, exhibiting a 94% survival rate. The acclimatized plants exhibited normal development with functional leaves and no morphological abnormalities.

## 3. Discussion

### 3.1. In Vitro Cutting Rooting

According to our previous study [9], the presence of IBA and the reduction of the MS medium to half strength were found to be more efficient in terms of the studied rooting traits and this explains why it was used as the only rooting hormone and as the exclusive medium. Moreover, the best values in *C. sativa* L. rooting traits, according to Lata et al. [33], were achieved using IBA. Once more, the presence of IBA was found to lead to significantly higher rooting traits of *C. sativa* L. [31,32]. This promoting effect of IBA on in vitro rooting performance has also been reported in several medicinal plants by Ferreira and Handro [51], Aminah et al. [52], Nadeem et al. [53], Zygomala et al. [54], and Patel and Shah [55]. Additionally, Caplan et al. [56], emphasized the stimulating effects of IBA in *C. sativa* L. stem cuttings, delivering a two-fold higher rooting success rate of 71% (maximum value) and higher root quality. Campbell et al. [57], when propagating hemp stem cuttings, reported rooting success using IBA concentrations that varied from 35% to 100%. We took advantage of IBA’s promotive effects to obtain the best possible results.

Many researchers have also reported the beneficial effect of the MS basal medium strength dilution on root initiation in *C. sativa* L. [58], *Typhonium flagelliforme* (G. Lodd.) Blume [59], *Camellia sinensis* L. [60], *Mentha spicata* L. [61] and *Mentha arvensis* L. [62], *Zingiber officinale* Roscoe [63], and *Syzygium alternifolium* (Wight) Walp. [64]. The increased rooting performance obtained using IBA has been documented in other species, i.e., *Rosa centifolia* L. and *Dodoneae viscosa* L., propagated using cuttings [65,66]. On the contrary, Movahedi et al. [67] stated that the longest root was formed in MS medium containing NAA. Conversely, Ślusarkiewicz-Jarzina et al. [58] reported root induction on MS basal medium containing IAA and NAA, whereas Smýkalová et al. [68] achieved root induction on growth-regulator-free medium or medium supplemented with NAA.

Even though the rooting percentages among the different treatments, in the two *C. sativa* L. varieties, were relatively different, it was observed that the majority of cuttings produced healthy roots in every rooting treatment. This higher percentage was compared to those produced in solidified agar medium in a previous study [9]. The rooting percentages were significantly different among treatments, thus indicating the need to pay attention to the selection of the appropriate treatment. As a result, the use of rooting sponges was more successful, possibly allowing better aeration in the rhizosphere due to their porosity. These results were similar to those obtained in several in vitro studies that used solid porous substrates, i.e., rockwool, phenolic foam, and polyester fiber block. Matching high rooting frequency rates have also been reported by other researchers. Kodym and Leeb [42] observed a 97.5% rooting percentage of in vitro *C. sativa* L. shoot tip cuttings in rockwool within 3 weeks in glass vessels with passive ventilation, although there is no information available concerning the multiplication rate and plant acclimatization in field conditions. Wróbel et al. [69] developed a modified method for rapid hemp regeneration using nodal cuttings, obtaining results that are comparable to those reported in this study. This method, however, did not use rockwool blocks as a substrate. Moreover, the results of this study converged with those of Zarei et al. [70], who applied a similar in vitro propagation method using cuttings in rockwool blocks, using only the cuttings of similar size (ca 3 cm). They reported that the cutting length influenced the rooting percentage and the mean root length, i.e., the longer cuttings exhibited better performance. Similar results to ours were obtained in in vitro studies of banana (*Musa*) and *Cymbidium* plants [38], in which rooting establishment took place in both agar and rockwool. Equivalent results were found in *Eucalyptus citriodora* Hook. by Nagae et al. [40]. In that study, plantlets showed a 90.6% rooting rate in rockwool compared to gellan gum medium (93.8%), although both substrates lacked rooting plant growth regulators. Our results are in accordance with those of Lata et al. [71], even though our corresponding rooting values are somehow lower. The highest root induction was obtained in treatments with low or medium IBA concentration results, which are in accordance to those of Movahedi et al. [67]. Analogous results also arose from comparisons of different rooting components in the ex vivo propagation of *Olea europaea* L. [72]. The rooting percentage observed in rockwool was the highest compared to the other treatments. In contrast, İsfendiyaroğlu and Kacar [72] examining *Vitis champinii* Planch. found that the differences between rooting blocks and IBA applications were not statistically significant in terms of the rooting percentage. They found that the highest rooting percentage, 43.2%, was obtained on phenolic foam substrate supplemented with 30 ppm IBA.

In addition to the strength of the MS medium, the average number of roots and average root length per cutting were significantly affected not only by the concentration but also by the way in which the IBA was applied. In an earlier study [9], the presence of IBA was found to be more efficient in terms of the average root number per shoot in both half- and full-strength MS media. Concerning the average root length, the best values were obtained in the presence of NAA. However, statistically, these treatments did not differ significantly from those obtained using IBA. Lata et al. [33] have also reported the highest values in terms of the average number and length of roots in *C. sativa* L. using IBA. Moreover, Lata et al. [71] reported in another study that the best average root length was recorded in IBA treatments.

The use of peat-based sponges had advantageous effects on rooting traits, compared to other porous substrates, i.e., rockwool, phenolic foam, and polyester blocks, used in several species and culture conditions. The numbers of roots and the root lengths of *Spathifyllum* explants on rockwool or polyester fiber blocks were increased compared to those on gellan gum medium [73]. Tanaka et al. [74] also used rockwool and found mixed results in terms of the number of roots as well in the average root length, depending on the species. According to these authors, on rockwool, *Gentiana* × *hybridus* and *Aechmea fasciata* (Lindl.) Baker presented incremental root numbers, which is similar to our results. Moreover, using the same medium, *Asparagus officinalis* L. and *Fragaria* × *ananassa* (Duchesne ex Weston) Duchesne ex Rozier, showed lower and higher values, respectively. On the contrary, for *Vitis vinifera* L., *Caladium bicolor* (Aiton) Vent., *Chrysanthemum morifolium* (Ramat.) Hemsl. and the “Beatrix” cv. of *Gerbera*, the number of roots was not affected by the substrate [74]. Conflicting results were obtained by Teixeira da Silva et al. [38] concerning the number of roots and the root length in Cymbidium and Musa. These traits depended on the permeability of the film used in the vessels’ lids. An advantageous effect of rockwool in photoautotrophic micropropagation was observed for *Spathiphyllum* [75] as well. Conversely, Nagae et al. [40], who used vented and non-vented vessels, found that the rockwool substrate did not beneficially influence either the number of roots or the average root length of *Eucalyptus citriodora* Hook. Moreover, Campbell et al. [57] reported that rooting in hemp (in terms of frequency and time) was genotype-dependent.

### 3.2. In Vitro Cutting Growth

The promising effect of the use of a porous substrate, i.e., rockwool, phenolic foam, or polyester blocks, in terms of height growth has already been reported in the past [38,39,40,42,75,76,77]. In medicinal *C. sativa* L., after a 5 weeks of in vitro culturing of plants on rockwool blocks in forced-ventilated glass jars, 95% of them had grown newly well-developed shoot tips [42]. Nagae et al. [78] reported similar results in height growth using rockwool in vented and non-vented vessels. In all culture vessels, the heights of plantlets cultured on rockwool were greater than those of plantlets cultured on gellan gum medium, in a CO_2_-enriched environment. Furthermore, the plantlets’ fresh weight remarkably increased on rockwool and platelets were more vigorous and significantly larger compared to those cultured on a conventional sugar-contained agar medium in bottles. Similar results were also obtained by Tanaka et al. [39,73]. They reported that the maximum plant height in *Spathiphyllum* was achieved on rockwool and polyester fiber blocks. In addition, polyester fiber blocks showed better results, with statistically significant differences compared to other treatments. In *Cymbidium*, an orchid, rockwool was shown to be the most suitable supporting medium for plantlet growth [38]. Likewise, Kubota and Kozai [79] showed that the growth of potato (*Solanum tuberosum* L.) plants cultured in a multi-cell tray with rock-wool cubes placed in polycarbonate vessels with forced ventilation was greater than those cultured using a conventional (small) culture vessel with natural ventilation. Conversely, Nagae et al. [40] reported that the rockwool substrate did not beneficially influence the height growth of *Eucalyptus citriodora* Hook. Concerning *Musa* plants micropropagated in vitro, Teixeira da Silva [38] found that there were no significant differences in the growth parameters when comparing rockwool and agar-derived plantlets.

Any differences in rooting traits and height growth, along with the effect of the strength and the particular components of the culture medium [55], such as the hormones used and their concentrations [9,31,80], could probably be attributed to different varieties/cultivars [9]. Moreover, these variations may also depend on the cutting type and the plant’s physiological condition [54], and are additionally influenced by genotype [9].

### 3.3. Acclimatization

In vitro rooted cuttings were successfully acclimatized. A high survival rate was achieved whe applying a two-phase acclimatization protocol [9]. Two-step acclimatization was applied by Lata et al. [71] and Lata et al. [33] as well. New growth was observed within three to four weeks. These results were similar to those of Lata et al. [81]. As Lata et al. [31] stated, acclimatization was associated with the plant growth regulator used. They found that m-topolin positively affected hardening to a greater extent compared to IBA, leading to more vigorous roots. Successful acclimatization was described by Kodym and Leeb [42], using rockwool blocks as the substrate, combined with commercially fertilizers, within 3 weeks in glass vessels with passive ventilation. In another study using rockwool blocks, Teixeira da Silva et al. [38] acclimatized *Cymbidium* and *Musa* plants micropropagated in vitro, which were grown under two culture systems. Moreover, Chandra et al. [32] reported the hardening of all plantlets after four weeks. Movahedi et al. [67] and Ślusarkiewicz-Jarzina et al. [58] also reported the successful acclimatization of in vitro rooted plantlets, with similar survival rates to those of the present study.

The application of this alternative method can lead to full aseptic propagating material after a period of only 3–4 weeks of culture. According to Kodym and Leebthe [42], the establishment of stock plants can be accomplished in 9 weeks and then their cuttings need about 5 additional weeks to become rooted and well-developed. In vitro shoot tip cuttings acclimatized within 3 weeks and the whole procedure was completed in 17 weeks. In this study, our plantlets were ready after 4 weeks, i.e., 2–3 weeks of rooting and growth in the vessels and 1–2 weeks of acclimatization. In vitro micropropagation always requires more time. Wang et al. [80] reported fully acclimatized plantlets after at least 12 weeks. Lata et al. [71] obtained hardened plantlets after 15–21 weeks, depending on treatments. In another study by Lata et al. [81] accomplished the whole process after 10 weeks. Zarei et al. [70] reported photoautotrophic micropropagation, as characteristically mentioned, of explants (cuttings) of different lengths (4, 5, and 7 cm) in a timeframe comparable to our results.

Additionally, the current in vitro propagation protocol of the selected H_CBD and H_CBG varieties results in the reduction of propagation costs. The method does not involve the use of agar or the subsequent manipulations that it requires, i.e., boiling the nutrient media. There is no use of cytokinins, thus decreasing the risk of somaclonal variation [35], and the use of sponges as the substrate is highly cost-saving. Furthermore, the rooted cuttings in the sponges are placed immediately for acclimatization, thus protecting the roots from being injured during the transfer of shoots for acclimatization and making the rooting process more efficient. In other cases, a solid nutrient medium is needed to wash the roots and remove the agar. After acclimatization, the plants are ready as independent units to be applied in industrial production. These latter two actions reduce the time required for hemp propagation. In addition, sponges absorb a certain amount of liquid nutrient medium, which they can engage, and there is the possibility of adding additional liquid nutrient medium if necessary. After inserting the cuttings in the sponges, they are put in the growth chamber until rooting is achieved. The plantlets do not require transportation from the growth chamber to dedicated acclimatization-hardening facilities or a special greenhouse. The lids are simply removed and the rooted plants are placed in the pots containing the peat–perlite mixture, leaving them in the same growth chamber. The use of this protocol could result in 500–600 rooted plantlets per 1 m^2^ of shelving area, thus enabling mass production.

## 4. Materials and Methods

### 4.1. Plant Material—Cutting Disinfestation

Cuttings were excised from selected healthy young medical *C. sativa* L. plants at the vegetative growth stage. Two varieties, a high-cannabidiol plant variety (H_CBD) and a high-cannabigerol plant variety (H_CBG), of *C. sativa* L. (Cannabaceae) were included in the present study, kindly provided by Ekati Alchemy Lab SL (Barcelona, Spain). The cuttings’ donor plants were grown in a greenhouse located at the Institute of Mediterranean and Forest Ecosystems Hellenic Agricultural Organization “Demeter” in Athens, Greece. Elite (based on chemical profile) female plants were used in the experiments. Mother plants were selected during a previous research study [9] and were maintained at the vegetative stage for a photoperiod of 18 h. All plants were kept indoors, under controlled environmental conditions at 27 °C ± 2 °C, having approximately 500 μmol m^−2^ s^−1^ photosynthetic photon flux density. Nodal segments with a height of ca. 3.5 cm, containing axillary buds as well as one to two leaves from the healthy female mother (donor) plants, were used as cuttings.

Cuttings were surface-disinfected through successive immersion in 1.0% NaOCl (*v*/*v*) (10% NaOCl, Fluka, Buchs, Switzerland) supplemented with 0.05% (*v*/*v*) Tween-20 (Fisher Bioreagents, Pittsburgh, PA, USA), with continuous stirring for 12 min, followed by immersion in 70% ethanol (*v*/*v*) for 45 s. Prior to disinfection, the cuttings were trimmed and the majority of the expanded leaf area was removed. Each immersion was followed by three rinses with sterile deionized water that lasted for three minutes. The disinfection process took place under sterile conditions, as did all the downstream handlings. After disinfection, cuttings’ dead leaves and tissues were removed and then cuttings were cut into desired lengths (ca. 3.5 cm) with sterile instruments and under aseptic conditions.

### 4.2. Culture System—Culture Establishment

Two culture systems were used. The first consisted of magenta vessels with non-vented closures (Sigma Aldrich, Merck KGaA, Steinheim am Albuch, Germany), with each including four rooting peat-based sponges (Growth Technology, Taunton, UK) impregnated in 30 mL of culture medium. The sponge material was derived from naturally composted fine-milled sphagnum peat moss, and they were fully biodegradable. The second culture system comprised vessels with vented closures (Duchefa Biochemie, Haarlem, The Netherlands) and eight rooting sponges impregnated in 65 mL of culture medium. The sponges were trimmed so that they acquired the same shape and size, i.e., 3.0 cm × 3.0 cm × 2.0 cm. In order to minimize the effect of any residuals of fulvic or humic acids remaining in the sponges, they were washed carefully three times with deionized-distilled water. In order to remove such residues even more efficiently during washing, the sponges were cut in half, achieving another reduction in costs. Moreover, with the aim of eliminating the effect, if any, of the mentioned acids on the cuttings’ rooting and growth performance, we used controls in our experimental design. The structure of the sponges enabled the proper passage of air for root development, due to their porosity. All the components of the culture system (rooting sponges, medium, and vessels) were simultaneously sterilized via autoclaving at 121 °C and under 122 kPa of pressure for 20 min. Cuttings, after disinfection, were placed in the culture system, one cutting per sponge.

### 4.3. Culture Medium

Murashige and Skoog (MS) [82] medium was used as a nutritional medium, supplemented with 3% sucrose. The MS medium contained 3.12 mM potassium phosphate monobasic, 53.28 μM glycine, 1.12 μM nicotinic acid, 4.86 pyridoxine HCl, and 3 μM thiamine HCl instead of 1.25 mM, 26.64 μM, 0.56 μM, 2.43 μΜ, and 0.30 μM, respectively. The pH of the medium was adjusted to 5.8 with 0.1 N NaOH and 0.1 N HCl. The medium was supplemented with the following antibiotics—100 mg l^−1^ penicillin and 50 mg l^−1^ kanamycin in order to avoid bacterial and fungal contamination. Both antibiotics present antibacterial activity and for the latter, its antifungal activity was recently discovered and therefore it can be used in agriculture, in addition to medicine [83].

### 4.4. Medium Strength—Plant Growth Regulators—Growth Conditions

Half-strength modified MS medium was used as a nutrient medium. Three different sets of experiments were performed. The first concerned MS medium being supplemented with indole-3-butyric acid (IBA) at various concentrations (2.46, 4.92, and 9.84 µM), plus a control medium, i.e., without IBA, for comparisons. Then the mixture was sterilized via autoclaving at 121 °C and 122 kPa for 20 min, before adding it in the sterilized container including the sterilized sponges. The second set of experiments was similar to the first one but differed in the IBA sterilization method, i.e., the IBA was sterilized using a syringe filter and then added to the autoclaved medium under aseptic conditions. The third set of experiments involved dipping the cuttings’ basal tip in a rooting aqueous solution of IBA at 15.0 μM. Cuttings for control treatment were not dipped in the rooting hormone solution. After the dipping and control treatments, the cuttings were transferred to the culture system for rooting. The treatments used in all the experiments are shown in Table 4.

All cultures were incubated in a growth chamber at 23 °C ± 1 °C with a 16 h/8 h (light/dark) photoperiod, under cool-white fluorescent lamps of 50 μmol m^−2^ s^−1^ photosynthetic photon flux density at culture level.

### 4.5. Evaluation of Culture System

Root and cutting development were assessed in both culture systems, after a two-month culture period of the healthy cuttings. The effect of the various concentrations of plant growth regulators, the variety, the vessel type, and the interaction between the mentioned factors on the rooting percentage (%) and the number and length of roots per cutting protruding from the sponges were evaluated. Cutting growth was evaluated as well. For each treatment, eight cuttings in three replications were used. The experiments were arranged in a growth chamber in a completely randomized design.

### 4.6. Acclimatization

Vigorous plantlets in sponges with well-developed roots were transplanted into 6.5 cm × 6.5 cm × 8.0 cm plastic pots containing a 1 peat:2 perlite (*v*/*v*) sterile mixture. The pots were placed in 48 cm × 33 cm × 20 cm mini plastic greenhouses (BHR, Pont-Pean, France) with plastic covers in order to avoid water loss and maintain humidity. All the plantlets were kept under controlled environmental conditions at 25 °C ± 2 °C with a 16 h/8 h (light/dark) photoperiod, with an approximately 50 μmol m^−2^ s^−1^ photosynthetic photon flux density at culture level. The plantlets were irrigated individually every day, if necessary, with tap water and the walls of the mini plastic greenhouses were sprayed with water to maintain adequate moisture. Acclimatization was achieved by gradually opening the plastic front door of the mini plastic greenhouses. The acclimatized plantlets were transplanted to flowerpots and placed indoors, under controlled environmental conditions at 25 °C ± 2 °C with a 12 h fluorescent photoperiod, with an approximately 500 μmol m^−2^ s^−1^ photosynthetic photon flux density, from flowering until maturity.

### 4.7. Statistical Analysis

Analysis was based on individual values of height growth, the percentage of rooted cuttings, the number of roots per cutting protruding from sponges, and the average length of roots per treatment, which were assessed at a significance level of *a* = 0.05. The following linear model was used in the analysis to determine the influence of the variety, the vessel type, the IBA treatment, and the interaction between the variety and the vessel type. as well as between the variety and the IBA treatment:

y_ijkm_ = μ + v_i_ + c_j_ + t_k_ + v_i_*c_j_ + v_i_*t_k_ + e_ijkm_

where *y_ijkm_* is the phenotypic measurement for a trait of the *m*th cutting, the *k*th IBA treatment, the *j*th container (vessel type) and the *i*th variety, as dependent variables; *μ* is the fixed population mean of all cuttings; *v_i_* is the fixed effect of the *i*th variety; *c_j_* is the fixed effect of *j*th vessel type; *t_k_* is the random effect of the *k*th IBA treatment; v_i_*c_j_ is the interaction of the *i*th variety with the *j*th vessel type; v_i_*t_k_ is the interaction of the *i*th variety with the *k*th treatment; and *e_ijkm_* is the random residual error of the *m*th cutting, the *k*th IBA treatment, the *j*th vessel type, and the *i*th variety. The variance components were estimated by means of the restricted maximum likelihood (REML) method. Descriptive statistics, analysis of variance (ANOVA), as well as the Duncan’s multiple range test (MRT) based on the 0.05 level of significance were performed on height growth, the percentage of rooted cuttings, and the number of roots per cutting protruding from sponges, as well as the average length of roots per treatment. Data, expressed as percentages, were subjected to appropriate log or arcsine transformation for analysis of their proportions before statistical analysis and were transformed back to percentages for presentation in tables and graphs. All statistical analysis was performed using SPSS v.20 software for Windows (IBM SPSS Statistics 2011, IBM Corp., Armonk, NY, USA).

## 5. Conclusions

An efficient alternative in vitro propagation protocol was developed for the large-scale production of the two selected H_CBD and H_CBG varieties. Using peat-based sponges and liquid media, we eliminated any deficiencies caused by root washing, in order to remove the agar, during solid media use, before acclimatization, as well as providing sufficient cutting support. Moreover, sponges absorb a certain amount of liquid nutrient medium, which engages the sponge. If necessary, the culture system may be supplemented with an additional amount of medium, depending on the conditions. This process is not only time-consuming, but can damage the fine roots and increase the possibilities of infection as well. Furthermore, in our proposed method the plantlets are better prepared for the final step of acclimatization, as rooted cuttings in sponges are immediately transferred for acclimatization, presenting increased growth and reduced mortality, since their roots are already well-formed, thus increasing hardening efficiency.

Overall, the best rooting traits for both varieties were achieved when supplementing the nutrient media via filter sterilization, at 4.92 μM, or by dipping the basal part of the cuttings in 15 mM aqueous solution. The use of vented vessels was more appropriate for H_CBD, whereas the non-vented vessels were more suited to H_CBG. Dipping treatments are easier to apply and their implementation does not require specialized staff. These regeneration methods were not only proven to enable savings in time and cost, but also demonstrated a high root formation frequency, as well as a high survival rate. The evolved regeneration culture system for *C. sativa* L. plants can be useful not only for propagating plant material at an industrial scale but also in projects aiming at genetic conservation and the preservation of selected varieties that are rich in CBD, CBG, THC, and other minor and rare cannabinoids, focusing on the improvement of the selected varieties. It was found that variations in root induction, root traits, and height growth were genotype-dependent.

## 6. Patents

A provisional patent application has been submitted to the Hellenic Industrial Property Organization (OBI). Furthermore, due to the its applicability to the international scientific community, the patent is also pending before the World Intellectual Property Organization (WIPO) according to the Patent Cooperation Treaty (PCT).

## Figures and Tables

**Table 1:** The effect of IBA treatments and concentrations on the average number and length of roots per cutting and the rooting percentage of the high-CBD *Cannabis sativa* L. variety.

Vessel Type	Treatment Concentration		Average Root Number per Cutting	Average Root Length (cm)	Average Rooting Percentage (%)
Non-vented	IBA autoclaved with the medium	2.46 μM	3.56 ^fgh^	0.73 ^cde^	66.67 ^bc^
		4.92 μM	6.29 ^de^	1.02 ^bc^	87.50 ^ab^
		9.84 μM	4.35 ^fg^	0.72 ^cde^	70.83 ^bc^
	IBA filter-sterilized	2.46 μM	2.97 ^gh^	1.54 ^a^	79.09 ^abc^
		4.92 μM	7.18 ^cd^	1.51 ^a^	87.50 ^ab^
		9.84 μM	4.79 ^fg^	0.86 ^cd^	79.17 ^abc^
	Dipping treatment 15 mM IBA	2 min	8.75 ^ab^	0.66 ^ef^	83.33 ^ab^
		4 min	9.47 ^a^	0.57 ^ef^	79.17 ^abc^
		6 min	5.53 ^ef^	0.71 ^cde^	79.17 ^abc^
	Control		2.62 ^h^	0.63 ^ef^	54.17 ^c^
Vented	IBA autoclaved with the medium	2.46 μM	2.85 ^h^	0.64 ^ef^	72.22 ^bc^
		4.92 μM	3.56 ^fgh^	0.53 ^ef^	88.89 ^ab^
		9.84 μM	3.08 ^gh^	0.58 ^ef^	72.22 ^bc^
	IBA filter-sterilized	2.46 μM	3.69 ^fgh^	1.21 ^b^	100.00 ^a^
		4.92 μM	4.56 ^fg^	0.97 ^bc^	94.44 ^ab^
		9.84 μM	3.00 ^gh^	0.65 ^ef^	72.22 ^bc^
	Dipping treatment 15 mM IBA	2 min	7.59 ^bcd^	0.64 ^ef^	94.44 ^ab^
		4 min	8.27 ^bc^	0.55 ^ef^	93.75 ^ab^
		6 min	4.07 ^fgh^	0.58 ^ef^	77.78 ^abc^
	Control		3.10 ^gh^	0.50 ^f^	55.56 ^c^

IBA: indole-3-butyric acid, Control: no IBA addition. Means followed by the same letter do not differ statistically at *p* ≤ 0.05 according to the Duncan test.

**Table 2:** The effect of IBA treatments and concentrations on the average number and length of roots per cutting and rooting percentage of the high-CBG *Cannabis sativa* L. variety.

Vessel Type	Treatment Concentration		Average Root Number per Cutting	Average Root Length (cm)	Average Rooting Percentage (%)
Non-vented	IBA autoclaved with the medium	2.46 μM	3.76 ^f^	0.74 ^abcd^	70.83 ^bcd^
		4.92 μM	5.13 ^de^	0.86 ^ab^	95.83 ^a^
		9.84 μM	3.43 ^f^	0.70 ^bcd^	87.50 ^abs^
	IBA filter-sterilized	2.46 μM	3.86 ^f^	0.81 ^abc^	87.50 ^abc^
		4.92 μM	5.26 ^cd^	0.88 ^a^	95.83 ^a^
		9.84 μM	4.05 ^ef^	0.71 ^bcd^	87.50 ^abc^
	Dipping treatment 15 mM IBA	2 min	6.90 ^ab^	0.83 ^abc^	83.33 ^abc^
		4 min	7.79 ^a^	0.77 ^abc^	79.17 ^abc^
		6 min	5.88 ^bcd^	0.62 ^ef^	66.67 ^bcd^
	Control		3.29 ^f^	0.47 ^gh^	58.33 ^de^
Vented	IBA autoclaved with the medium	2.46 μM	3.36 ^f^	0.65 ^cde^	61.11 ^cde^
		4.92 μM	3.88 ^f^	0.60 ^efg^	94.44 ^ab^
		9.84 μM	3.64 ^f^	0.59 ^fg^	77.78 ^abc^
	IBA filter-sterilized	2.46 μM	3.63 ^f^	0.72 ^bcd^	88.89 ^ab^
		4.92 μM	4.19 ^def^	0.68 ^cd^	88.89 ^ab^
		9.84 μM	3.29 ^f^	0.66 ^cde^	77.78 ^abc^
	Dipping treatment 15 mM IBA	2 min	6.24 ^bc^	0.72 ^bcd^	94.44 ^ab^
		4 min	6.75 ^ab^	0.61 ^efg^	88.89 ^abc^
		6 min	6.42 ^bc^	0.49 ^gh^	66.67 ^bcd^
	Control		3.00 ^f^	0.37 ^h^	50.00 ^de^

IBA: indole-3-butyric acid, Control: no IBA addition. Means followed by the same letter do not differ statistically at *p* ≤ 0.05 according to the Duncan test.

**Table 3:** The effects of IBA treatments and concentrations on cutting growth of the high-CBD plants and high-CBG *Cannabis sativa* L. varieties.

Type of Vessel	Treatment Concentration (μΜ)	Length (cm)		
		High-CBD Plants		High_CBG Plants
Non-vented	IBA autoclaved with the medium	2.46 μM	0.82 ^cde^	0.98 ^cde^
		4.92 μM	1.42 ^ab^	1.50 ^ab^
		9.84 μM	1.14 ^bcd^	1.11 ^bcde^
	IBA filter-sterilized	2.46 μM	0.95 ^cde^	1.23 ^abcd^
		4.92 μM	1.59 ^a^	1.60 ^a^
		9.84 μM	1.18 ^bc^	1.12 ^bcde^
	Dipping treatment 15 mM IBA	2 min	1.43 ^ab^	1.34 ^abc^
		4 min	0.71 ^def^	0.83 ^def^
		6 min	1.12 ^bcd^	0.75 ^ef^
	Control	No PGR	0.61 ^efg^	0.73 ^ef^
Vented	IBA autoclaved with the medium medium	2.46 μM	0.64 ^ef^	0.84 ^def^
		4.92 μM	1.17 ^bc^	1.13 ^bcd^
		9.84 μM	0.72 ^def^	0.85 ^def^
	IBA filter-sterilized	2.46 μM	0.82 ^cde^	1.00 ^cde^
		4.92 μM	1.31 ^ab^	1.24 ^abc^
		9.84 μM	1.03 ^cd^	0.87 ^def^
	Dipping treatment 15 mM IBA	2 min	0.73 ^def^	1.18 ^bcd^
		4 min	0.52 ^g^	0.96 ^cde^
		6 min	0.65 ^ef^	0.96 ^cde^
	Control	0.54 ^fg^		0.57 ^g^

IBA: indole-3-butyric acid, Control: no IBA addition. Means followed by the same letter do not differ statistically at *p* ≤ 0.05 according to the Duncan test.

**Table 4:** The treatments used in the experiments. The used medium was the half-strength MS.

Variety	Treatment Concentration		
	IBA Autoclaved with the Medium	IBA Filter Sterilized	Dipping Treatment 15 mM IBA
High-CBD plants	Control	Control	Control
	2.46 μM	2.46 μM	2 min
	4.92 μM	4.92 μM	4 min
	9.84 μM	9.84 μM	6 min
High-CBG plants	Control	Control	Control
	2.46 μM	2.46 μM	2 min
	4.92 μM	4.92 μM	4 min
	9.84 μM	9.84 μM	6 min

## Data Availability

Data not available.
